# Longitudinal Surveillance of Porcine Rotavirus B Strains from the United States and Canada and *In Silico* Identification of Antigenically Important Sites

**DOI:** 10.3390/pathogens6040064

**Published:** 2017-12-03

**Authors:** Frances K. Shepherd, Fangzhou Chen, Marie R. Culhane, Michael P. Murtaugh, Douglas G. Marthaler

**Affiliations:** 1 Department of Veterinary and Biomedical Sciences, College of Veterinary Medicine, University of Minnesota, 1971 Commonwealth Avenue, St. Paul, MN 55108, USA; sheph085@umn.edu (F.K.S.); murta001@umn.edu (M.P.M.); 2 State Key Laboratory of Agricultural Microbiology, College of Veterinary Medicine, Huazhong Agricultural University, Wuhan 430070, China; chenfangzhou@webmail.hzau.edu.cn; 3 Department of Veterinary Population Medicine, College of Veterinary Medicine, University of Minnesota, 1365 Gortner Avenue, St. Paul, MN 55108, USA; grame003@umn.edu; 4 Veterinary Diagnostic Laboratory, Kansas State University, 1800 Denison Ave, Manhattan, KS 66506, USA

**Keywords:** rotavirus B virus, antigenic epitopes, vaccine, genetics, swine

## Abstract

Rotavirus B (RVB) is an important swine pathogen, but control and prevention strategies are limited without an available vaccine. To develop a subunit RVB vaccine with maximal effect, we characterized the amino acid sequence variability and predicted antigenicity of RVB viral protein 7 (VP7), a major neutralizing antibody target, from clinically infected pigs in the United States and Canada. We identified genotype-specific antigenic sites that may be antibody neutralization targets. While some antigenic sites had high amino acid functional group diversity, nine antigenic sites were completely conserved. Analysis of nucleotide substitution rates at amino acid sites (dN/dS) suggested that negative selection appeared to be playing a larger role in the evolution of the identified antigenic sites when compared to positive selection, and was identified in six of the nine conserved antigenic sites. These results identified important characteristics of RVB VP7 variability and evolution and suggest antigenic residues on RVB VP7 that are negatively selected and highly conserved may be good candidate regions to include in a subunit vaccine design due to their tendency to remain stable.

## 1. Introduction

Rotaviruses (RVs) cause acute diarrhea in suckling and weaned pigs worldwide. Members of the Reoviridae family, RVs contain 11 segments of double stranded RNA that encode six structural viral proteins (VPs) and five or six nonstructural proteins (NSPs) [1]. Rotaviruses have a triple layer capsid, with an outer capsid that is comprised of the VP7 and VP4, which stimulate neutralizing antibodies in the host [1]. The VP4 protein is cleaved into the VP5* and VP8*, and the VP5* and VP7 mediate entry into the cell. Serologic and genetic characteristics of the VP6 (inner capsid) are used to classify rotavirus species, and ten RV species (RVA-RVJ) have been discovered [1,2,3]. Within each RV species, sequencing or the antigenic properties of the VP7 and VP4 designates the G (Glycoprotein) and P (Protease-sensitivity) genotypes and serotypes, respectively.

RVA was the first rotavirus species that was discovered as an enteric virus in cattle [4,5] and later in pigs in the 1970s. In the 1980s, “atypical” rotaviruses were detected in swine and were subsequently classified as Rotavirus B (RVB) [6,7,8]. Other host species for RVB include humans [9], cattle [10], sheep [11], goats [12], and rats [13]. To date, porcine RVB has been detected in the United States, Canada, Mexico, Brazil, India, Germany, and Japan [14,15,16,17,18]. Porcine RVB studies demonstrated that RVB detection in clinical samples increases with animal age [16,19]. Given an unexpectedly high identification of RVB within the United States (47% of porcine clinical samples in 2009), a focus on preventive measures for swine is warranted [16]. 

An 80% nucleotide identity cutoff value has established 21 RVB G genotypes, while a P genotype classification scheme is not yet established for RVB [16]. In RVA studies, vaccination with one genotype does not provide reliable heterotypic protection against other RVA genotypes [20,21], making it necessary for vaccines to contain strains that are homologous or closely related to those on the farm. Subunit vaccines contain primarily antigenic viral regions with a wide range of genetic diversity to match the RV strain on the farm [22,23]. In general, subunit vaccines eliminate the need for culturing the pathogen and reduce the risk of vaccine reversion to virulence. While a single report demonstrated that RVB could be adapted to cell culture [24], RVB is very difficult to grow and propagate in culture, and a modified live RVB vaccine is lacking, leading to an interest in subunit vaccines to prevent piglet mortality.

In mouse models, subunit vaccines for RVA utilizing VP8* were able to elicit high neutralizing antibody titers [25,26]. Extending this vaccine research to RVB VP7 is a significant step in developing control methods for rotavirus infections in swine. However, an understanding of the antigenicity and variability of the virus strains is necessary in order to choose the best candidate gene segments for a subunit vaccine. In order to further these efforts and since wet laboratory experiments are lacking to describe the neutralizing epitopes of RVB, a longitudinal sampling of RVB strains from the United States and Canada was used to identify variable regions, predict antigenicity, and perform analysis of positive and negative selection on RVB VP7.

## 2. Results

Porcine intestinal or fecal samples that were submitted to the University of Minnesota Veterinary Diagnostic Laboratory between 2011 and 2017 were analyzed for RVB using RT-qPCR. Samples with positive RVB detection were sent for sequencing of the VP7 gene (*n* = 174). Samples originated from the United States (*n* = 159) and Canada (*n* = 15). The RVB strains MN-125, MN-126, MN-127, and IA-79 contained a three nucleotide insertion at position 105, resulting in a larger open reading frame (747 to 750 nucleotides). 

**Figure 1:**
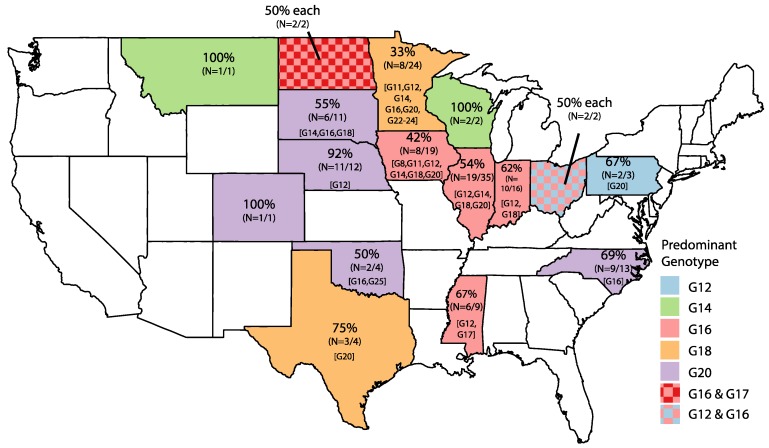
Distribution of RVB genotypes by state. States are colored according to dominant G genotype, and the percentage of the dominant G genotype is represented in the top line of each state. Number of strains belonging to dominant genotype is indicated in parentheses out of the total strains from that state. Brackets indicate additional G genotypes identified in the state.

Eight RVB G genotypes were identified: G8 (*n* = 1), G11 (*n* = 2), G12 (*n* = 15), G14 (*n* = 11), G16 (*n* = 68), G17 (*n* = 3), G18 (*n* = 17), and G20 (*n* = 52) (Table 1). Five American strains had nucleotide percent identity values of below 80% when compared to all of the strains in GenBank (NCBI), and were assigned new genotypes of G22 (strain MN-98), G23 (strains MN-125 and MN-126), G24 (strain MN-127), and G25 (strain OK-63). The G16 genotype was the most prevalent genotype each year. The greatest genotype diversity occurred in 2013 and 2014, when eight genotypes were identified. United States strains originated from pigs in 16 states (Figure 1). The predominant genotypes (G12, G14, G16, G18, and G20) clustered geographically, with G12 being predominant on the east coast, G16 in the Midwest, and G20 within the Great Plains states.

**Figure 2:**
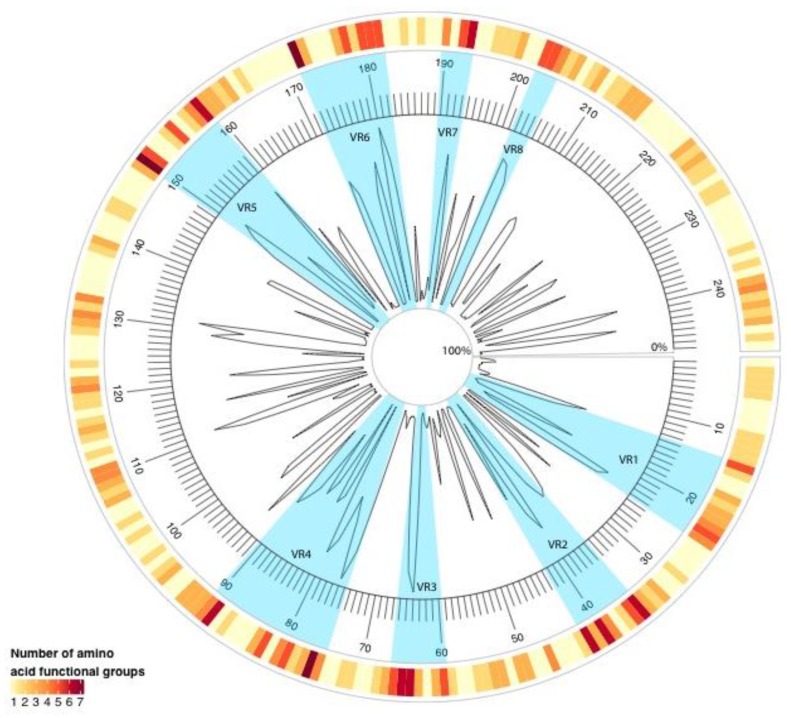
Radial line graph of mean amino acid pairwise percent identity of RVB VP7 sequences. The outer circle heatmap represents the number of amino acid functional groups present at each residue position. The inner circle denotes amino acid residue locations 0 through 248. Blue sections highlight the variable regions (VRs).

To investigate the variability within RVB VP7 proteins, the mean amino acid pairwise percent identity and the number of amino acid functional groups were calculated at each residue. As shown in Figure 2, residues 76, 150, and 172 had seven different amino acid functional groups in all of the RVB G genotypes. Additionally, eight variable regions (VRs) were identified (VR1, at amino acid positions 14–24; VR2, 35–42; VR3, 60–66; VR4, 75–90; VR5, 150–159; VR6, 172–182; VR7, 190–193; and, VR8, 202–204).

The RVB VP7 strains were characterized by comparing the ratio of nucleotide substitutions at synonymous and non-synonymous sites dN/dS analysis to find residues undergoing positive and negative selection. Analysis of all the RVB strains revealed 11 positions (2, 5, 10, 14, 20, 23, 64, 65, 159, 178, and 244) with significant positive selection by one or more methods (Table S1). Interestingly, the majority of these sites were only identified by the MEME (Mixed Effects Model of Evolution) method, which tests for episodic rather than pervasive positive selection [27]. Analysis of the five predominant genotypes indicated that episodic selection identified in the whole RVB genotypes was only present in certain genotypes, such as sites 64 and 65, which were positively selected only in G16 strains. 

**Figure 3:**
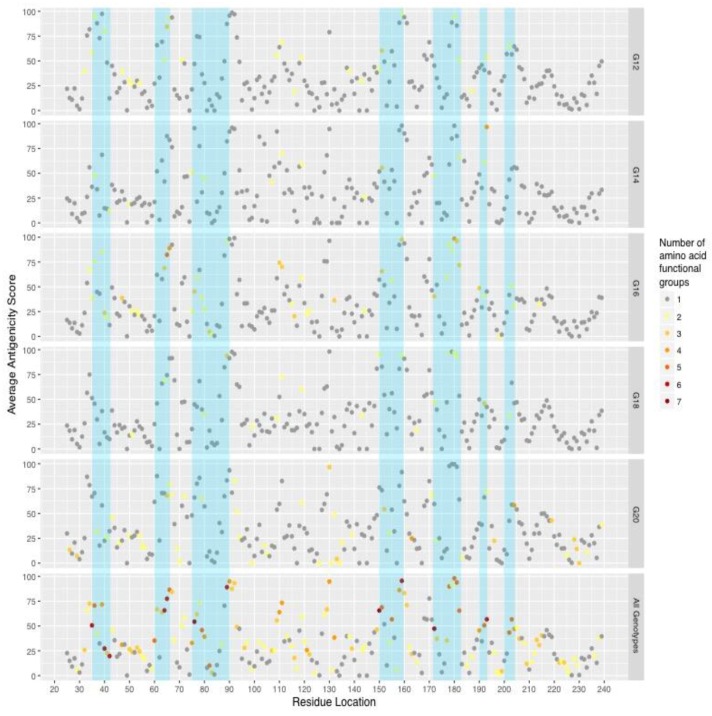
Average predicted antigenicity within predominant genotypes and their relationships with variable regions (blue).

To determine if variability may translate to antigenic escape, EPCES (Epitope Prediction by ConsEnsus Scoring) [28] was used to predict antigenic residues with protein models of RVB VP7 created by homology modeling with a crystallized RVA VP7 structure. The average Global Model Quality Estimation (GMQE) score for the RVB models was 0.47. Deletion of the single amino acid insertion at site 36 in strains MN-125, MN-126, MN-127, and IA-79 did not affect the protein conformation or antigenic predictions (data not shown). Antigenicity scores were averaged across all of the RVB G genotypes and in the five predominant genotype groups (Figure 3). Highly antigenic sites were not consistent across the predominant genotypes. The G12 genotype group had a cluster of six highly antigenic sites between residues 33 and 40, while G16, G18, and G20 genotypes had a single or two antigenic sites within the same residue positions. G14 had no highly antigenic sites.

Since sites of high antigenicity did not consistently align with VRs, we investigated genotype-specific amino acid functional diversity at antigenic residues. Four categories were generated: complete functional conservation across the five major genotypes (*n* = 9 sites), high within-genotype conservation (functional groups conserved within each genotype, *n* = 8 sites), moderate within-genotype conservation (functional groups conserved within three or four genotype groups, with one or two groups varying, *n* = 11 sites), and variability across all of the genotypes (*n* = 10 sites) (Table 2). The functional groups of 19 of the 20 amino acids were represented in the antigenic sites, with the exception of cysteine. Although 10 antigenic sites exhibited high variability across all predominant genotypes, residue location 36 maintained similar side chain volume and hydropathic properties while residue locations 65 and 66 retained polarity according to PRIME analysis (PRoperty Informed Models of Evolution, http://hyphy.org/w/index.php/PRIME), which detects conserved and non-conserved amino acid properties.

**Figure 4:**
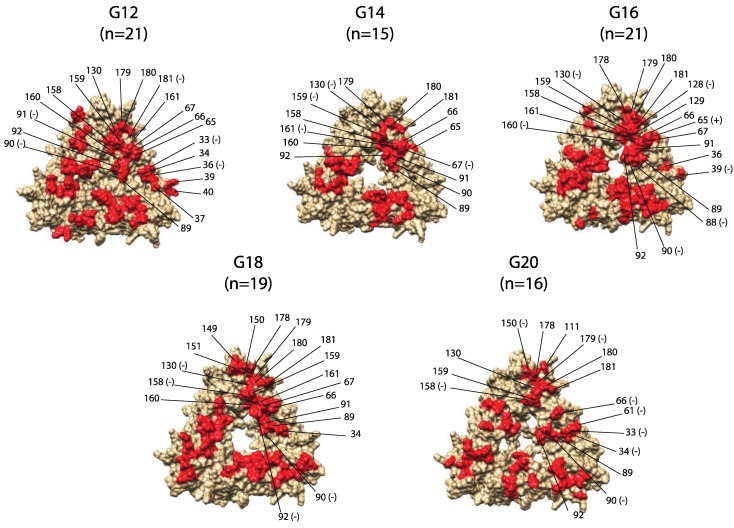
Genotype-specific predicted antigenic sites on RVB VP7 trimer. Residues are labeled with (+) if positively selected or (−) if negatively selected, and are labeled for one monomer of the trimer, modeled from a strain of the respective genotype.

Protein modeling highlighted surface-exposed antigenic sites for possible vaccine targets (Figure 4). Eight of 38 predicted antigenic sites were in locations inaccessible to antibody binding, while G18 had an additional residue (88) inaccessible to antibody binding (Table 2). Surface-exposed antigenic residues underwent negative selection more often than positive selection. Negative selection was also detected in several antigenic sites with conserved amino acid functional groups (sites 61, 67, 158, 160, 161, and 179).

## 3. Discussion

Swine RVB is a pathogen of interest throughout the United States and across the globe. In 2009, G16 and G20 genotypes were the most dominant in the United States [16], which is consistent with our more recent findings. The four newly discovered G22–G25 genotypes indicates high diversity of RVB when compared to other RV species, especially in swine where 20 RVB G genotypes have been identified when compared to the 12 RVA genotypes [29]. Although inoculation of RVB in gnotobiotic piglets causes clinical disease [7], RVB more commonly exists as a co-infection with RVA and RVC in conventional piglets [19,30], thus raising the question regarding the role of RVB as a primary or secondary pathogen. If RVB is not a primary pathogen, evolutionary pressures from the host immune system may facilitate unrestricted evolution into novel genotypes. Non-pathogenic hantaviruses [31] and *Fusarium oxysporum* [32,33] have higher genetic diversity when compared to their pathogenic relatives, which supports the hypothesis of RVB as a secondary pathogen.

Our study bioinformatically predicted antibody epitopes on RVB VP7, which is a significant step toward developing vaccination strategies for the pathogen. The amino acid variability at the identified epitopes ranged from complete conservation to high variation, suggesting that analysis of variability may not be a reliable predictor of epitopes in RVB, as it is in other RV species. In human RVA G3 strains, for instance, a correlation was found between locations of lineage-specific amino acid variation and known neutralization epitopes [34]. Using bioinformatic methods to predict antibody epitopes has limitations. Defining why surface-exposed amino acids are recognizable by antibodies is difficult, and wet lab experiments may not comprehensively identify all of the epitopes on a protein. These issues make it difficult to train epitope prediction algorithms to perform well on novel proteins, as previous studies have discussed [35]. Inaccurate epitope prediction was most clearly observed in our dataset, where 8 out of 38 (21%) antigenic sites that were predicted by EPCES were inaccessible to antibody binding, which is near the 30% of false positivity rate with this algorithm [28]. We attempted to correct for inaccuracies by only choosing amino acid residues with very high antigenic scores (above 75).

The small number of crystallized rotavirus proteins that were available to use as templates for predicting the protein structure of RVB limits our analysis. As indicated by the low GMQE scores, RVA is not the best template for RVB and may affect antigenic site predictions. Future work should focus on crystallizing structures of RVB for more accurate analysis of antibody binding sites, and monoclonal antibody experiments are necessary to confirm epitopes in RVB. Moreover, while B cell and antibody epitopes are important for pathogen neutralization, T cells are highly involved in processing and presenting antigens. The inclusion of a universal T cell antigen may help to stimulate a more robust immune response to a RVB subunit vaccine, and should be explored further [25].

Amino acid biochemical properties of epitopes drive antibody-antigen interactions [36]. In an H5 influenza A virus (IAV) antibody binding site, a functionally conserved amino acid change from valine to leucine resulted in minimal change in antibody binding affinity [37]. Conversely, mutations changing charge, structure, or hydrophobicity can significantly interrupt the binding of monoclonal antibodies to IAV [37,38]. Although variability at antigenic sites poses a challenge for vaccine development, many biochemical properties of amino acids at predicted epitopes on RVB VP7 were maintained, and an RVB vaccine that is designed to target these conserved properties might be more broadly neutralizing. Amino acids near epitopes can play a role in antibody binding and neutralization, as in HIV, where a conservative functional change from isoleucine to valine on an epitope on the gp41 protein unexpectedly resulted in greater neutralization [39]. Therefore, monoclonal antibody experiments using RVB are necessary to fully elucidate the efficacy of designing vaccines based on functional amino acid characteristics. The predicted epitopes of the predominant genotype groups lacked cysteine residues, which also was observed in epitopes of RVA VP7 [40,41]. Conservation of all the cysteine residues in our RVB dataset suggest that they maintain a stable VP7 trimer in RVB, as was shown for RVA [42].

Sites of genotype-specific positive selection suggest that the evolution of RVB VP7 may be genotype dependent. Locations of positive selection in hepatitis B vaccine escape mutants differed for the different genotypes as well [43]. In our dataset, positive selection was often only identified by one method and was sometimes contradicted by other methods that predicted negative selection at the same site, such as at position 66 in G18 strains (Supplementary Table S1). Therefore, reported positive selection sites should be interpreted with discretion. Antigenic sites are typically surface-exposed and are predicted to benefit from positive selection, which might facilitate immune escape. However, our results suggest that negative selection is acting more strongly in the evolution of the RVB VP7 antigenic sites. Generally, RNA viruses have higher rates of purifying selection when compared to DNA viruses [44]. From a functional perspective, motifs on RVA VP7 are known to be involved with binding to host cell integrins to facilitate entry [45,46,47,48], and some of the RVB surface-exposed and stable residues may be important for host cell recognition since RVB VP7 genotypes are host-specific [16]. Negative selection is known to play a role in the evolution of neutralizing epitopes in other viruses as well, such as poliovirus [49]. Identification of several antigenic sites that had completely conserved amino acid functional groups was surprising and should be incorporated into a subunit vaccine.

In conclusion, the genetic analysis of RVB VP7 strains identified new G genotypes and potential residues important in immune interactions. Antigenic sites under negative selection may be better vaccine candidates due to their propensity for synonymous change. Future research should include analysis of the differences in variability and antigenicity of the VP4 protein of RVB, which is another target of neutralization, and in vivo experiments to confirm our findings and understanding of neutralizing epitopes for RVB.

## 4. Materials and Methods

The University of Minnesota Veterinary Diagnostic Laboratory received porcine intestinal or fecal samples for diagnostic testing. Samples were determined positive for RVB by real time RT-qPCR, and the positive RVB samples were sequenced based on requests from the veterinarian [19]. Open reading frames for VP7 were generated, and genotypes were assigned using BLAST (NCBI), and an 85% nucleotide identity cutoff value [16]. Genotype distribution was mapped using ggplot and maps packages in R, and Adobe Illustrator. The VP7 sequences were translated and aligned using MUSCLE in Geneious (v9.6.1, www.geneious.com) [50]. Sequences were submitted to Genbank (MF522263-MF522436).

Indels were deleted from the alignment to compare residues that were present in all strains. A mean amino acid pairwise percent identity value was generated by comparing all of the pairs of amino acids within a residue and adding a score of 1 each time that the strain was identical to another. The sum of the scores within a residue was divided by the total number of residues.

The number of functional amino acid groups per residue was calculated. Functional amino acid groups were defined as nonpolar, aliphatic carbon chains (Ala, Gly, Ile, Leu, and Val); nonpolar, aliphatic, sulfur-containing chains (Met); nonpolar, aromatic (Phe and Trp); nonpolar, cyclic (Pro); polar hydroxyl-containing (Ser and Thr); polar amides (Asn and Gln); polar sulfur-containing (Cys); polar, aromatic (Tyr); acidic (Asp and Glu); basic, aliphatic (Lys and Arg); and, basic, cyclic (His). Mean amino acid pairwise percent identity and functional diversity were visualized using the Circlize package in R [51]. Variable regions (VRs) were identified by amino acid diversity using a ten residue sliding window, and defined if three or more locations had greater than three functional amino acid groups represented. VRs were smaller than the window size if the 10th residue location did not have four functional groups.

Monomers of RVB VP7 were generated in SWISS-MODEL (https://swissmodel.expasy.org/) using a crystallized structure of a rhesus RVA strain (PDB 3fmg) as the template to generate protein structures as input for Epitope Prediction by ConsEnsus Scoring (EPCES, http://sysbio.unl.edu/EPCES/index.php) and predict the RVB VP7 antigenic epitopes. EPCES utilizes six aspects to predict the antigenic residues: residue epitope propensity, conservation score, side-chain energy score, contact number, surface planarity score, and composition [28]. Each of the predominant genotypes were submitted individually to EPCES, and values greater than 75 were considered highly as antigenic for each amino acid site. Visualization of predicted antigenic sites used UCSF Chimera (https://www.cgl.ucsf.edu/chimera/). The protein structures underwent energy minimization to eliminate protein structure clashing and small geometry distortions. Coloring and labeling of trimer structures was done in Chimera and Adobe Illustrator.

Comparisons of synonymous and non-synonymous nucleotide substitution rates of VP7 utilized the SLAC (Single-Likelihood Ancestor Counting), FUBAR (Fast Unconstrained Bayesian AppRoximation), FEL (Fixed Effects Likelihood), and MEME (Mixed Effects Model of Evolution) methods in DataMonkey (www.datamonkey.org) [27,52,53,54]. The significance threshold for SLAC was set at 0.1, due to its more conservative estimation of selected sites. The two-tailed posterior probability threshold for FUBAR was set at 0.9. The p-value threshold for FEL and MEME were set at 0.05. All of the algorithms identified pervasive positive selection while MEME also identified episodic positive selection, or selection that was only present in a subset of the data. PRIME analysis (PRoperty Informed Models of Evolution, http://hyphy.org/w/index.php/PRIME), implemented in DataMonkey, was used to identify changing and conserved biochemical properties at the antigenic sites based on the Conant-Stadler properties of amino acids (chemical composition, polarity, volume, iso-electric point, and hydropathy) [55].

## Figures and Tables

**Table 1:** Distribution of Rotavirus B (RVB) G genotypes by year.

Genotype	Year							
	2011	2012	2013	2014	2015	2016	2017	Total Sequences
**G8**	0	0	1	0	0	0	0	**1**
**G11**	0	0	1	0	1	0	0	**2**
**G12**	3	4	2	2	3	1	0	**15**
**G14**	1	0	1	2	3	4	0	**11**
**G16**	9	9	19	18	6	5	2	**68**
**G17**	0	2	0	0	0	1	0	**3**
**G18**	0	7	2	5	1	2	0	**17**
**G20**	0	8	19	17	2	5	1	**52**
**G22**	0	0	0	1	0	0	0	**1**
**G23**	0	0	0	2	0	0	0	**2**
**G24**	0	0	0	1	0	0	0	**1**
**G25**	0	0	1	0	0	0	0	**1**
**Total Sequences** **(Genotypes)**	**13** **(3)**	**30** **(5)**	**46** **(8)**	**48** **(8)**	**16** **(5)**	**18** **(6)**	**3** **(2)**	**174**

**Table 2:** Amino acid variability of highly antigenic residues across the 5 predominant genotypes

Residue Location	G12 [*n* = 21]	G14 [*n* = 15]	G16 [*n* = 21]	G18 [*n* = 19]	G20 [*n* = 16]	Changed Properties	Conserved Properties
33	D ^−^	D	D	D	N ^−^		
34	D	D	N	N	D/N ^−^		
36	T/N ^−^	Q/T	N	N	N/T	Chemical composition	Volume, hydropathy
37	D	E	E	E	E		Iso-electric point
39	K	K/R	T ^−^	K	R/T/K		Chemical composition
40	K/Q	E	Q/E	E	E		
61	N	N	N	N	N ^−^		
65	N/E/D	E/D	Q/R^+^	D	D/N	Volume	Polarity
66	N/D	N	N/S/D	N	N/K ^−^		Polarity
67	Y	Y ^−^	Y	Y	Y		
76 *	H	Y ^−^	T/I/V/M	Y	N/S		Iso-electric point
77 *	N	N	D/N	D	N/S		
78 *	Y	Y	Y	Y	F ^−^		
88 *	V	V	V/I ^−^	V	I		Hydropathy
89	K	A	D	S/G/D	D/N		
90	Y ^−^	D	E ^−^	D ^−^	D ^−^		Iso-electric point
91	A ^−^	P	P	P	P		Polarity
92	Y	Y	W	F ^−^	W		
102 *	E	E ^−^	E	E	Q		
104 *	N	N	N	N	N		
109 *	A/T	V/A	A	A/T	A		
111	G/R	K/N	N	E/D/N	E/G		
128	S	S	S ^−^	S	A		
129	R/K	K	K/R	K/R	M/T	Polarity	Volume, iso-electric point, hydropathy
130	D	G ^−^	G ^−^	G ^−^	D/N		
149	L	L	L	T	T/I		Iso-electric point
150	E/K	S	S	Y	P ^−^	Polarity	Chemical composition
151	G/D	G/S/A/N	S/D/G	T	G/T/E/S		
158	P	P	P	P ^−^	P ^−^		
159	D/N	K ^−^	E/K	E/D	N/K/D/S		Volume
160	R	R	R ^−^	R	R		
161	R	R ^−^	K	R	K/R		
170 *	F	F	F ^−^	F ^−^	F ^−^		
178	Y	Y	H	R	R	Polarity	
179	S	S	S	S	S ^−^		
180	N	D/E	T/A/K	S	Q/E		Iso-electric point
181	N ^−^	D	S/N	H/Y	N/S		
193 *	Q/P	L/D/N	P	P	P		

Red text indicates the residue location is antigenic for the given genotype. Positive and negative selection indicated by (+) and (−), respectively. Functional diversity at each residue locations highlighted according to complete conservation (green), high within-genotype conservation (blue), moderate within-genotype conservation (yellow), or variability across all genotype groups (pink). Asterisks (*) indicates sites inaccessible to antibody binding (antigenic site at residue 88 is inaccessible in G18 strains only). Brackets indicate number of predicted surface-exposed neutralizing epitopes per genotype.

## Supplementary Materials

The following are available online at www.mdpi.com/2076-0817/6/4/64/s1.
