# Supplementary material for: Longitudinal Surveillance of Porcine Rotavirus B Strains from the United States and Canada and In Silico Identification of Antigenically Important Sites
Source: Pathogens. 2017 Dec 3;6(4):64. doi: 10.3390/pathogens6040064 (PMC5750588; doi:10.3390/pathogens6040064)
Supplement: Supplementary file 1 [file pathogens-06-00064-s001.pdf]

Supplementary Material:

Supplementary table S1. Positively selected sites for the 5 predominant and all VP7 genotypes. Positive selection (green) or negative selection (red) highlighted per genotype, with identifying method listed. NS= neutral selection.

Codon	G12	G14	G16	G18	G20	All Strains
**2**	FEL, FUBAR	NS	NS	NS	NS	MEME
**3**	NS	NS	MEME	MEME	NS	NS
**5**	NS	NS	NS	NS	NS	FUBAR, MEME
**10**	NS	NS	FEL, FUBAR	FUBAR	MEME	MEME
**14**	NS	MEME	NS	MEME	MEME	MEME
**20**	FUBAR	NS	NS	NS	MEME FEL and FUBAR	MEME
**21**	FUBAR	FUBAR	NS	FEL, FUBAR	MEME FUBAR	FEL, FUBAR, SLAC
**22**	FUBAR	FUBAR	FEL, FUBAR, SLAC	NS	MEME	FEL, FUBAR, SLAC
**23**	NS	FUBAR	NS	NS	NS	SLAC, MEME
**39**	NS	FUBAR	FEL, FUBAR, SLAC	NS	MEME	FEL, FUBAR
**64**	FEL, FUBAR	NS	MEME	NS	FEL, FUBAR, SLAC	MEME
**65**	NS	NS	All	FEL, FUBAR, SLAC	FUBAR	MEME
**66**	NS	NS	NS	FUBAR	MEME FEL FUBAR and SLAC	FEL, FUBAR, SLAC
**83**	NS	FEL, FUBAR, SLAC	FEL, FUBAR, SLAC	MEME	FEL, FUBAR, SLAC	FEL, FUBAR, SLAC
**151**	FUBAR	NS	NS	FEL, FUBAR	MEME	NS
**159**	NS	FUBAR, SLAC, FEL	MEME	FEL, FUBAR	NS	MEME
**178**	FUBAR	FUBAR, SLAC, FEL	FUBAR, SLAC	NS	MEME	MEME
**180**	FEL, FUBAR	NS	MEME	MEME	NS	NS
**244**	NS	NS	NS	NS	MEME	MEME
